# Expression of Microbial Enzymes in Mammalian Astrocytes to Modulate Lactate Release

**DOI:** 10.3390/brainsci11081056

**Published:** 2021-08-10

**Authors:** Barbara Vaccari Cardoso, Iliana Barrera, Valentina Mosienko, Alexander V. Gourine, Sergey Kasparov, Anja G. Teschemacher

**Affiliations:** 1 School of Physiology, Pharmacology and Neuroscience, University of Bristol, Bristol BS8 1TD, UK; b.vaccaricardoso@bristol.ac.uk (B.V.C.); iliana.barrera34@gmail.com (I.B.); Sergey.Kasparov@bristol.ac.uk (S.K.); 2 Institute of Biomedical and Clinical Sciences, College of Medicine and Health, University of Exeter, Exeter EX4 4PS, UK; V.Mosienko@exeter.ac.uk; 3 Centre for Cardiovascular and Metabolic Neuroscience, Department of Neuroscience, Physiology & Pharmacology, University College London, London WC1E 6BT, UK; a.gourine@ucl.ac.uk

**Keywords:** astrocytes, lactate, gliotransmitter, viral vectors

## Abstract

Astrocytes support and modulate neuronal activity through the release of L-lactate. The suggested roles of astrocytic lactate in the brain encompass an expanding range of vital functions, including central control of respiration and cardiovascular performance, learning, memory, executive behaviour and regulation of mood. Studying the effects of astrocytic lactate requires tools that limit the release of lactate selectively from astrocytes. Here, we report the validation in vitro of novel molecular constructs derived from enzymes originally found in bacteria, that when expressed in astrocytes, interfere with lactate handling. When lactate 2-monooxygenase derived from *M. smegmatis* was specifically expressed in astrocytes, it reduced intracellular lactate pools as well as lactate release upon stimulation. D-lactate dehydrogenase derived from *L. bulgaricus* diverts pyruvate towards D-lactate production and release by astrocytes, which may affect signalling properties of lactate in the brain. Together with lactate oxidase, which we have previously described, this set of transgenic tools can be employed to better understand astrocytic lactate release and its role in the regulation of neuronal activity in different behavioural contexts.

## 1. Introduction

The glycolysis product L-lactate (lactate) in the brain has been receiving increasing attention as a growing number of studies suggest its roles in the modulation of neuronal circuit function as diverse as memory, decision making, response to environmental novelty, depression, central arousal, or autonomic regulation [1,2,3,4,5,6,7]. Likewise, where the mechanisms of lactate actions have been investigated, these, too, are diverse and are discussed in the context of meeting metabolic demands by intercellular energy substrate shuttling, or receptor-mediated signalling, or modulation of gene expression [4,8,9,10,11].

Astrocytes can be considered the main source of brain extracellular lactate since they are endowed with the machinery to import glucose from the periphery, store energy in the form of glycogen, and dynamically shuttle energy substrates throughout the astrocytic network and to the extracellular space [12]. Various studies have suggested that astrocytes favour the glycolytic pathway over mitochondrial oxidative phosphorylation, resulting in excess production and release of lactate [13]. The concentration gradient of lactate, as demonstrated for the cortex, is directed from the astrocytic towards the neuronal compartment [7,14].

In spite of considerable progress and interest in the subject, many questions remain open. As an intermediate product in equilibrium-based enzymatic processes that exits its cells of origin in a gradient-driven but also partially regulated manner, the flux of lactate through the cellular brain compartments in vivo has proven difficult to match up quantitively with its specific actions on cellular targets and brain circuits. We, therefore, aimed to develop a set of molecular tools to support studies into the roles of astrocyte-derived lactate in the brain. The rationale was to harness the activity of bacterial enzymes that metabolise lactate, and to express them in astrocytes. Our aim was to decrease intra-astrocytic lactate pools that can be released to the extracellular space, constitutively or in response to local signals, and consequently to reduce lactate action. We recently reported that viral vector-mediated expression of bacterial lactate oxidase (LOx) in hippocampal astrocytes modulates behaviour in the context of environmental novelty in mice [5]. Here we expand the novel toolset by two further constructs, lactate 2-monooxygenase (LMO) and D-lactate dehydrogenase (DLDH) and compare the properties between these and LOx when expressed in rodent astrocytes.

**Figure 1:**
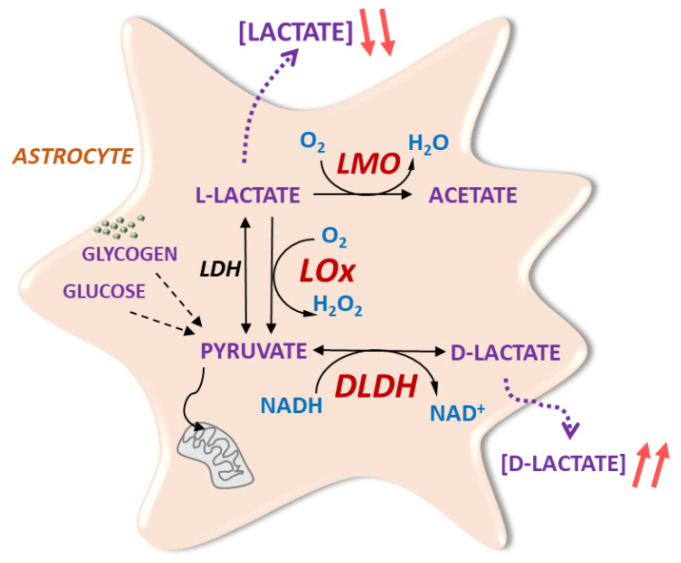
Novel molecular tools based on bacterial enzymes to modulate astrocytic lactate signalling. Astrocytic lactate dehydrogenase (LDH) converts pyruvate from glycolytic activity to lactate. Lactate is irreversibly oxidised by transgenic LMO of microbial origin to acetate, or to pyruvate by LOx (as previously described in [5]). This decreases the intracellular lactate pool and limits lactate release from astrocytes. Bacteria-derived DLDH reversibly catalyses the conversion of pyruvate into D-lactate, which is subsequently released from astrocytes.

LMO, like LOx, irreversibly oxidises lactate into pyruvate at the expense of flavin cofactor reduction, which in turn is re-oxidised by molecular oxygen in a 2-step reaction [15]. In LOx, pyruvate is released very rapidly from the enzymatic complex, a feature that accounts for the fast reaction kinetics of this enzyme [15]. As a result, molecular oxygen, which functions as an electron acceptor, forms hydrogen peroxide. LMO activity, on the other hand, exhibits higher kinetic stability, which makes pyruvate dissociation from the enzymatic complex a significantly slower process [15]. Thus, the reaction of the pyruvate-enzymatic complex with molecular oxygen culminates in pyruvate decarboxylation, yielding acetate and water (Figure 1). We investigated potential differences in the time course of action and reactive oxygen species-related effects as these criteria could favour the use of one tool over the other for specific applications.

DLDH catalyses the reversible stereospecific NAD-dependent conversion of pyruvate into D-lactate (DL; Figure 1). This was predicted to raise the DL/lactate ratio in astrocytes and, subsequently, in the extracellular space. For many lactate-dependent processes, DL is a weak substrate and may therefore indirectly impact on lactate-dependent processes but, in certain scenarios, e.g., lactate action in the *locus coeruleus*, DL can act as lactate antagonist [4].

Here we demonstrate the enzymatic activity of viral vector-based expression of LMO and DLDH in rat astrocytes in vitro and suggest that the use of these constructs may facilitate future studies into the functional roles of lactate in the CNS.

## 2. Materials and Methods

### 2.1. Vector Construction

**Figure 2:**
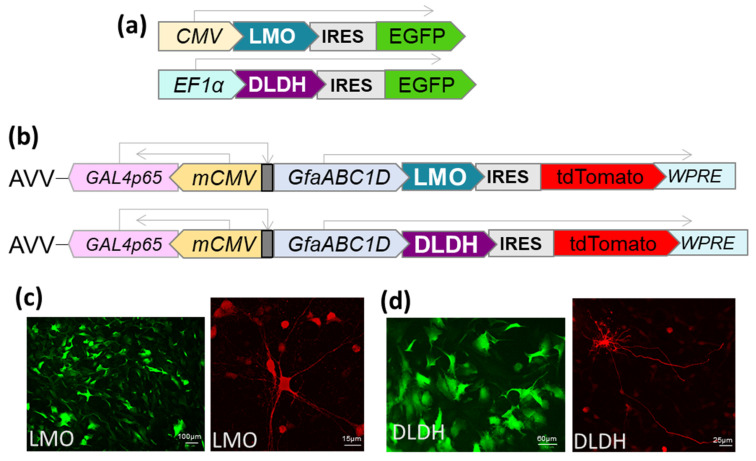
Expression of LMO and DLDH in mammalian cells. (**a**) Domain structure of the cassette for transfection of HEK293 cells with LMO and DLDH. The expression was driven by CMV or EF1α promoter. Enhanced green fluorescent protein (EGFP) was cloned 3’ of an internal ribosome entry site (IRES). (**b**) A transcriptionally enhanced fragment of GFAP promoter (sGFAP, [16]) was employed to selectively target the expression of the enzymes to astrocytes. The bicistronic cassettes were introduced into an AVV backbone with tdTomato as a fluorescent marker. (**c**) Representative images of astrocytes in dissociated primary culture (left; scale bar 100 µm) or organotypic brainstem slices (right; scale bar 15 µm) transduced with AVV-sGFAP-LMO-IRES-tdTomato. (**d**) Representative images of astrocytes in dissociated primary culture (left; scale bar 60 µm) or organotypic brainstem slices (right; scale bar 25 µm) transduced with AVV-sGFAP-DLDH-IRES-tdTomato. The reporter was amplified by anti-RFP staining.

The LMO (E.C. 1.13.12.4) coding sequence from *Mycobacterium smegmatis* (GenBank: AIU22323.1) and the DLDH (E.C. 1.1.1.28) sequence from *Lactobacillus delbrueckii* subsp. *bulgaricus* (GenBank: WP013438907) were optimised for mammalian codon usage and synthesised by Invitrogen (Life Technologies, Paisley, UK). Expression cassettes were cloned to be driven by either CMV, EF1-α or the astrocyte-selective transcriptionally-enhanced sGFAP promoter [16]. In addition, an internal ribosomal entry site (IRES), followed by a fluorescent marker (EGFP or tdTomato), was included downstream of the LMO or DLDH sequence (Figure 2). The cassettes were transferred to an adenoviral vector (AVV) shuttle plasmid (pXCX) and vectors derived from type 5 adenovirus were produced as described previously [17]. For experiments in HEK293, expression plasmids were transfected using TransIT-293 (Mirus, MIR 2700).

To enable assessment of changes in intracellular lactate or pyruvate, AVVs for CMV-driven expression of the FRET-based reporters for lactate and pyruvate, Laconic and Pyronic, respectively, were used [5,18,19].

### 2.2. Astrocyte Cultures

Wistar rat pups were sacrificed in accordance with Schedule 1 of the UK Home Office (Scientific Procedures) Act (1986) and as approved by the University of Bristol ethics committees. Primary dissociated astrocytes and organotypic slice cultures were prepared as previously described [5,20], and AVVs were added to the culture media. The multiplicity of infection (MOI) was determined from the AVV titer (transforming units/ml) as a ratio between transforming units added and cell count per culture well. Organotypic cultures were transduced with AVV at the time of plating and used 8–12 days later; dissociated cultures were transduced at least 2 days before experimentation.

### 2.3. Cell Viability Assays

For estimation of cell viability with the Trypan Blue exclusion assay, astrocytes were trypsinised, harvested, and stained with trypan blue 0.4% (Sigma-Aldrich, T8154, Dorset, UK). The percentage of blue non-viable cells in the suspension was determined in a hemocytometer. For the XTT cell viability assay (Cell Signaling Technology, 9095, Beverly, MA, USA), the formazan formation reaction was performed for 2 h. The relative metabolic viability of the cells was derived from the sample’s absorbance at 450 nm using a microplate reader (Tecan Infinite M200 PRO, Labtech, Heathfield, UK).

### 2.4. Immunohistochemistry

Primary cultured astrocytes seeded on glass coverslips or brainstem organotypic slices excised from the culture membrane were fixed for 15 min in ice-cold 4% paraformaldehyde (PFA) in phosphate buffer (PBS; pH 7.4). After rinsing in PBS, the samples were incubated for 1 h at room temperature in a solution of 0.3% Triton X-100 (Sigma-Aldrich, T8787) and 10% serum. Samples were incubated overnight at 4 °C with anti-RFP antibody (Rockland Immunochemicals, 600-401-379S; 1:200, Limerick, PA, USA), rinsed in PBS and incubated for 1 h at room temperature with Alexa Fluor 594 antibody (Thermo Fisher Scientific, R37117, Waltham, MA, USA).

### 2.5. Western Blot

After 72 h of AVV transduction or 3 h of staurosporine (5 µM) treatment, respectively, astrocytes were washed twice with cold PBS, treated with RIPA buffer containing protease and phosphatase inhibitors (Sigma-Aldrich) and harvested. After lysis, cells debris was centrifuged at 12,000× *g* and frozen at −80 °C until use. Protein extracts were prepared for SDS-PAGE with sample buffer (NuPAGE™ LDS Sample Buffer 4×, Invitrogen) and reducing agent (NuPAGE™ Sample Reducing Agent 10×, Invitrogen), then heated for 5 min to 95 °C. Following protein quantification using Pierce™ BCA Protein Assay Kit (Thermo Fisher Scientific), equal amounts of protein were run on 10% acrylamide gel (NuPAGE™ Bis-Tris, Invitrogen), then transferred to a nitrocellulose membrane (Amersham™ Hybond™, Thermo Fisher Scientific) using Trans-Blot Cell (Bio-Rad, Watford, UK) and visualised with Ponceau staining (Sigma-Aldrich). Membranes were blocked with 2% of ECL blocking reagent in PBS-0.1% Tween 20 (PBS-T) for 1 h at room temperature, before being incubated overnight at 4 °C with the primary antibodies: LDHA (1:2000, Cell Signaling), LDHB (1:1000, Abcam, Cambridge, UK), or cleaved caspase 3 antibody (1:1000, Cell Signaling). The same membranes were later incubated with Actin antibody (1:20,000, Sigma). Following three 5 min washing steps in PBS-T, membranes were incubated for 1 h at room temperature with secondary HRP-linked antibodies: Swine anti-rabbit IgG HRP (1:10,000, Agilent, Stockport, UK) or goat anti-mouse IgG HRP (1:10,000, Dako). Enhanced Chemiluminescence EZ ECL kit (Geneflow Limited, Lichfield, UK) was used for immunodetection. Images were taken with G:BOX (Syngene, Cambridge, UK) operated with GeneSys 1.6.9 software. The quantification of immunoblots was performed with Image-J software. Total levels of protein were calculated as the ratio of the LDHA or LDHB with actin from the same membrane. 

### 2.6. Imaging of Intracellular Lactate and Pyruvate

Primary cultured astrocytes on glass coverslips expressing Laconic or Pyronic and either LMO or DLDH with tdTomato were transferred to a recording chamber on an upright confocal microscope (Leica SP1 or SP5) and superfused with HEPES-buffered solution (HBS; in mM: NaCl 137, KCl 5.4 or 3, Na_2_HPO_4_ 0.34, KH_2_PO_4_ 0.44, CaCl_2_ 1.6, MgSO_4_ 0.8, NaHCO_3_ 4.2, HEPES 10, Glucose 5.5 or 2; pH 7.4; 32.5 ± 1 °C). Laconic or Pyronic were excited with the 458 nm line of an Argon ion laser and the emission of mTFP and Venus was detected at wavelength 465–500 nm and 515–595 nm, respectively. Images were taken every 3 s. Regions of interest were selected and the FRET ratio (mTFP/Venus) was calculated and normalized to the baseline. 

### 2.7. Determination of Lactate and DL Release from Dissociated Cultures

Lactate or DL levels in conditioned media from dissociated cells expressing LMO or DLDH were determined with fluorometric assays (BioAssay Systems, EFLLC-100 and EFDLC-100, Hayward, CA, USA). HEK293 cells or astrocytes were exposed to serum-free culture media for 2 or 6 h, respectively. Samples used for quantification of DL underwent deproteination using a 10 kDa spin filter to deplete any potential activity of endogenous LDH enzyme. Samples were then processed according to the manufacturer’s instructions and fluorescence intensity was measured in a microplate reader (λ_excitation_: 530 nm, λ_emission_: 585 nm). Lactate and DL concentrations were calibrated to a standard curve.

### 2.8. Determination of Extracellular Lactate in Organotypic Brainstem Slices

Amperometric lactate biosensors (Sarissa Biomedical, SBS-LAC-05-50, SBI-NUL-05-50, Coventry, UK) were used for real-time recording of extracellular lactate dynamics in organotypic brainstem slices. Slices were transferred to a recording chamber continuously superfused with HBS (see above). The lactate electrode was placed in contact with the surface of the slice and allowed to stabilise for at least 30 min. The null electrode was positioned on the contralateral side of the slice and its readout was subtracted from the current generated by the lactate sensor.

The potential at the electrode surface was controlled by a potentiostat (Duo-Stat ME200+) and the signal was acquired and analysed using a 1401 interface and Spike 2 software (Cambridge Electronic Design, Cambridge, UK). Known amounts of lactate were applied to the chamber at the beginning and at the end of each recording to calibrate the electrodes.

### 2.9. Statistical Analysis

Comparison between two experimental groups was made using two-tailed *t*-tests, paired or unpaired, as indicated. Data from three or more groups were compared using ANOVA followed by post hoc Tukey’s test or non-parametric Wilcoxon Mann–Whitney U test, as indicated. The bars in the scatter plot graphs represent the mean and the error bars the standard deviation. Statistical tests were performed using GraphPad Prism software. Differences with *p* < 0.05 between the experimental groups were considered significant.

## 3. Results

### 3.1. Potential Negative Effects on the Viability of Astrocytes

**Figure 3:**
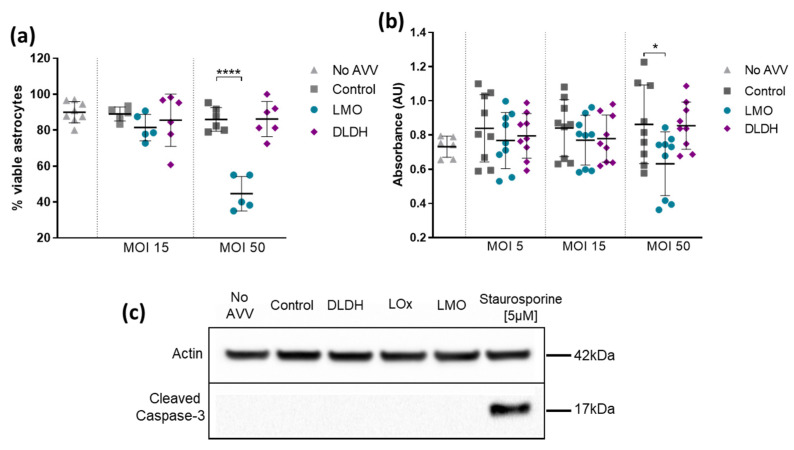
Viability of primary cultured astrocytes transduced with LMO and DLDH. Non-transduced astrocytes (No AVV) and astrocytes transduced with AVV-sGFAP-EGFP (Control), AVV-sGFAP-LMO-IRES-tdTomato (LMO), or AVV-sGFAP-DLDH-IRES-tdTomato (DLDH) at different MOIs were compared with regards to cell viability. (**a**) Trypan Blue exclusion assay; sample sizes *n* = 8 (No AVV), *n* = 5 (Control MOI15, LMO MOI15, LMO MOI50), *n* = 6 (Control MOI50, DLDH MOI15, DLDH MOI50). (**b**) XTT assay; sample sizes *n* = 6 (No AVV), *n* = 9 all other conditions. Cytotoxicity was observed only with transduction of LMO at MOI 50. **** *p* < 0.0001, * *p* < 0.05, one way ANOVA with Tukey multiple comparison test. (**c**) Absence of caspase 3 activation in primary astrocytes transduced with the novel tools as compared with astrocytes incubated with Staurosporine [5 µM] for 3 h.

The consequences of interfering with lactate metabolism or outcomes from the presence of new enzymatic reactions in astrocytes are hard to predict. Therefore, in order to identify any cytotoxic effects of the novel tools and to optimise the viral vector titres, primary cultured astrocytes were transduced with AVV and cell viability was assessed using XTT and Trypan Blue exclusion assays. AVV-sGFAP-DLDH-IRES-tdTomato and the control vector AVV-sGFAP-EGFP did not compromise cell viability in any MOI test (Figure 3). Transduction with AVV-sGFAP-LMO-IRES-tdTomato did not affect astrocytic viability below an MOI of 50 (Figure 3). All validation experiments in vitro were therefore carried out at MOI 15. Quantification of caspase 3 activation under these conditions showed an absence of pro-apoptotic effects of the AVVs on astrocytes (Figure 3c).

### 3.2. DLDH Expression Results in Constitutive DL Release

**Figure 4:**
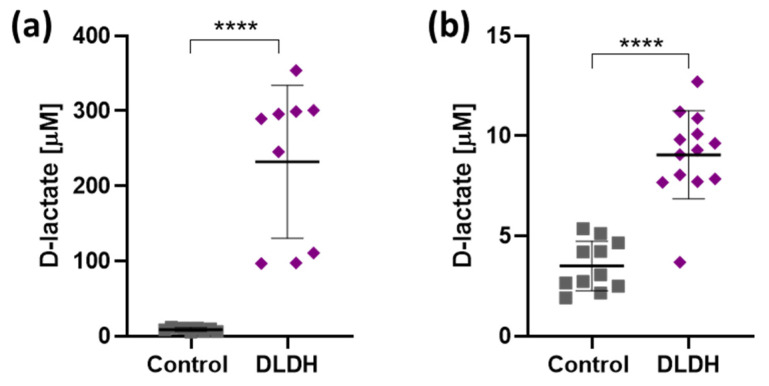
Constitutive D-lactate release from cells expressing DLDH. (**a**) Media conditioned by non-transfected HEK293 cells (Control, *n* = 9) and HEK293 cells transfected with 1.5 µg/µL of pTYF-EF1α-DLDH-IRES-EGFP (DLDH, *n* = 9). (**b**) Media conditioned by non-transduced cultured astrocytes (Control, *n* = 11) and astrocytes transduced with AVV-sGFAP-DLDH-IRES-tdTomato (DLDH, *n* = 13). **** *p* < 0.0001, unpaired two-tails *t*-tests.

The enzymatic activity of DLDH cells was assessed through analysis of DL content in conditioned media collected from DLDH-expressing cell cultures. HEK293 cells transfected with pTYF-EF1α-DLDH-IRES-EGFP showed a 25.8-fold rise in secreted DL (from 9 µM in control to 232 µM; Figure 4a). In primary cultured astrocytes transduced with AVV-sGFAP-DLDH-IRES-tdTomato, DL release increased by 2.6 times as compared to non-transduced astrocytes (from 3.5 µM in control to 9 µM; Figure 4b). This confirms that DLDH expressed in mammalian cells is functional and leads to constitutive DL release.

### 3.3. Expression of LMO Reduces Constitutive Lactate Release

**Figure 5:**
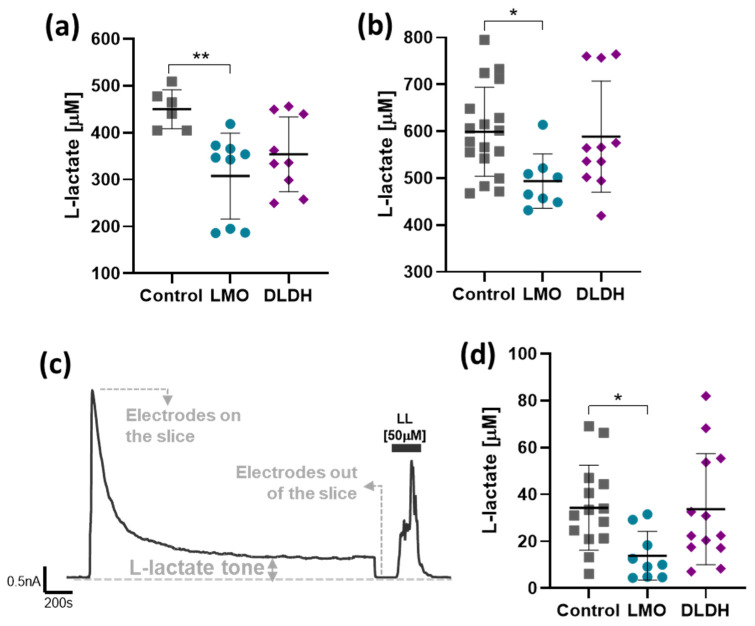
Effect of LMO or DLDH expression on constitutive lactate release. (**a**) Lactate concentration in media conditioned by HEK293 cells transfected with pCMV-IRES-EGFP (Control, *n* = 6), pCMV-LMO-IRES-EGFP (LMO, *n* = 9), or pTYF-EF1α-DLDH-IRES-EGFP (DLDH, *n* = 9). (**b**) Lactate concentration in media conditioned by primary cultured astrocytes transduced with AVV-sGFAP-EGFP (Control, *n* = 17), AVV-sGFAP-LMO-IRES-tdTomato (LMO, *n* = 8), or AVV-sGFAP-DLDH-IRES-tdTomato (DLDH, *n* = 11). ** *p* < 0.01, * *p* < 0.05, one way ANOVA with Tukey multiple comparison test. (**c**) Representative trace of an amperometric recording following contact and stabilization of the lactate sensor with the surface of the organotypic slice culture. (**d**) Extracellular lactate tone of organotypic cultures transduced with AVV-PRSx8-EGFP alone (Control, *n* = 14) or in combination with AVV-sGFAP-LMO-IRES-tdTomato (LMO, *n* = 9) or AVV-sGFAP-DHDH-IRES-tdTomato (DLDH, *n* = 13). * *p* < 0.05, Kruskal–Wallis one-way ANOVA on ranks, followed by Dunn’s test.

Lactate release from HEK293 cells transfected with pCMV-LMO-IRES-EGFP was reduced by 31.6% (Figure 5a), and a reduction of 17.6% was observed in primary cultured astrocytes transduced with AVV-sGFAP-LMO-IRES-tdTomato (Figure 5b). Expression of DLDH, on the other hand, did not significantly affect constitutive (L-)lactate release (Figure 5). 

The impact of the astrocyte-selective expression of LMO and DLDH on lactate tone was also assessed in organotypic slice cultures using amperometric lactate biosensors. Initial contact of the lactate biosensor with the surface of the slice resulted in a current spike which declined to a steady-state that reflected constitutive lactate release, i.e., the lactate tone (Figure 5c). Transduction of astrocytes in organotypic brainstem slices with AVV-sGFAP-LMO-IRES-tdTomato resulted in a significantly decreased lactate tone, whereas lactate tone in slices transduced with AVV-sGFAP-DLDH-IRES-tdTomato was not affected (Figure 5d).

### 3.4. LMO and DLDH Expression in Astrocytes Decreases Evoked Lactate Release

We next determined the effects of astrocytic expression of LMO and DLDH on the release of lactate upon acute stimulation. To force maximal lactate extrusion from cells, we stimulated monocarboxylate transporter (MCT) trans-acceleration by bath application of a high pyruvate concentration (10 mM), a well-established approach to deplete cells of lactate in vitro and in vivo [7,14,19]. By imposing a steep monocarboxylate (pyruvate) gradient, MCT activity is accelerated so that lactate is transported in the opposite direction and leaves the cells [21].

**Figure 6:**
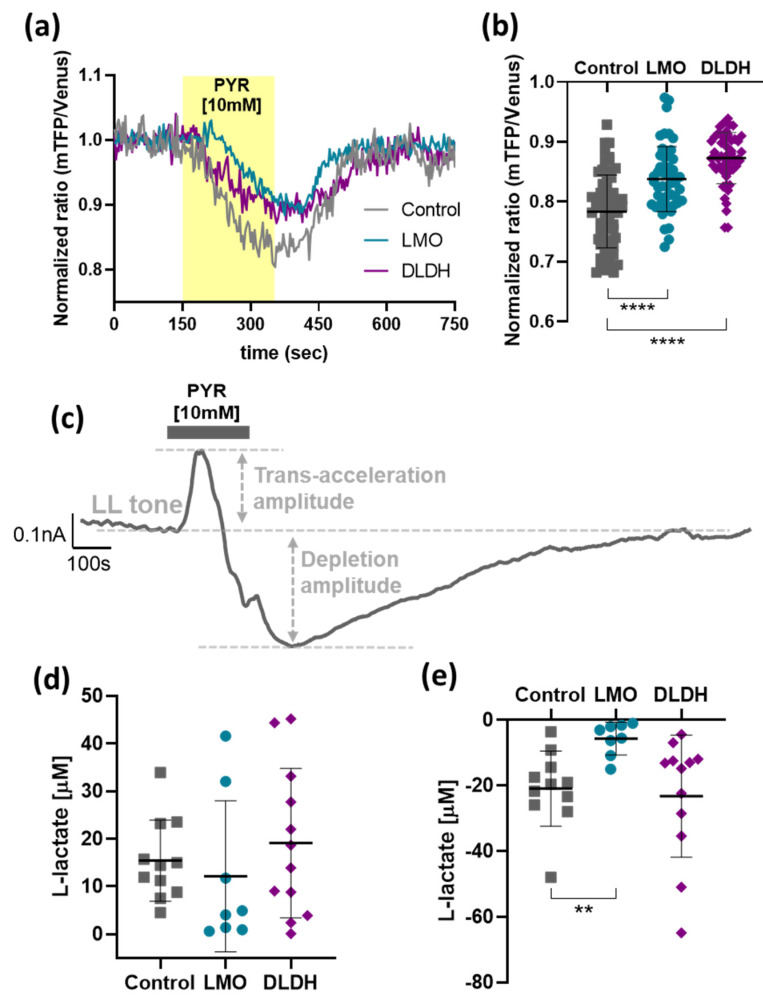
Effect of LMO and DLDH on the forced release of lactate. (**a**) Representative traces of Laconic FRET ratio following application of pyruvate [10 mM] in astrocytes expressing AVV-CMV-Laconic alone (Control) or in combination with AVV-sGFAP-LMO-IRES-tdTomato (LMO) or AVV-sGFAP-DLDH-IRES-tdTomato (DLDH). (**b**) Pooled data of the minimum value from Laconic FRET ratio following pyruvate application (Control, *n* = 62; LMO and DLDH, *n* = 52). **** *p* < 0.0001, one way ANOVA with Tukey multiple comparison test. (**c**) Representative trace of an amperometric recording during pyruvate [10 mM] application. (**d**) and (**e**) Pooled data for the trans-acceleration and depletion amplitude, respectively, following pyruvate [10 mM] application in organotypic brainstem slices transduced with AVV-PRSx8-EGFP alone (Control, *n* = 11) or in combination with either AVV-sGFAP-LMO-IRES-tdTomato (LMO, *n* = 8) or AVV-sGFAP-DLDH-IRES-tdTomato (DLDH, *n* = 12). ** *p* < 0.01, Kruskal–Wallis one-way ANOVA on ranks, followed by Dunn’s test.

Bath application of pyruvate evoked a substantial drop in the intracellular concentration of lactate in cultured astrocytes as reported by the FRET sensor Laconic (Figure 6a). Extracellular lactate levels measured using biosensors in organotypic brainstem slices increased transiently in response to pyruvate, indicating lactate release (Figure 6c). In dissociated astrocytes, trans-acceleration-driven lactate release was significantly decreased when cells were transduced to express either LMO or DLDH (Figure 6b). The net amplitude of trans-acceleration-driven lactate release from organotypic brainstem slices transduced to express LMO or DLDH in astrocytes exhibited a large variability, precluding the detection of any significant effect of LMO and DLDH (Figure 6d). Pyruvate-induced lactate release in organotypic brainstem slices was followed by a transient decrease in lactate tone, reaching a minimum that may reflect the transient depletion of intracellular lactate pools (Figure 6c; depletion amplitude). This may indicate that newly produced lactate is then released to the extracellular space at a reduced rate until intracellular lactate stocks are restored to their baseline levels, and the initial tone is re-established. The amplitude of lactate depletion triggered by pyruvate was reduced in organotypic brainstem slices containing LMO-expressing astrocytes (Figure 6e).

### 3.5. Expression of LMO or DLDH Does Not Affect LDH Activity in Astrocytes

**Figure 7:**
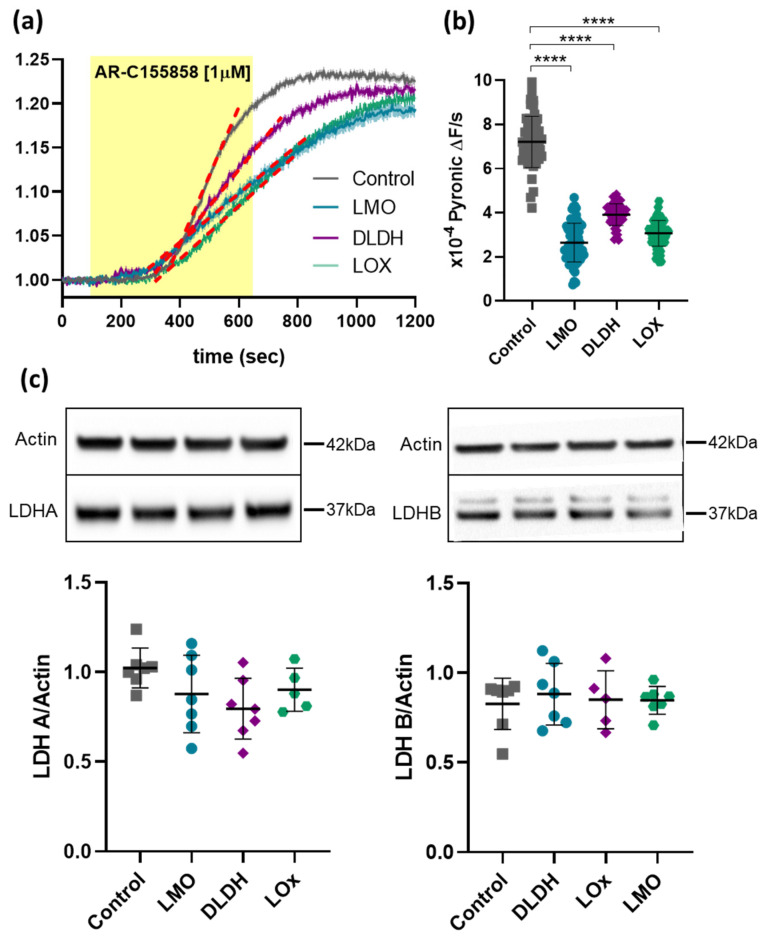
Intracellular pyruvate build-up upon blockade of MCT1. (**a**) Time course of the average Pyronic FRET ratio upon AR-C155858 [1 µM] application in primary cultured astrocytes transduced with AVV-CMV-Pyronic alone (Control, *n* = 52) or in combination with either AVV-sGFAP-LMO-IRES-tdTomato (LMO, *n* = 69), AVV-sGFAP-DLDH-IRES-tdTomato (DLDH, *n* = 53), or AVV-sGFAP-LOx-IRES-tdTomato (LOx, *n* = 72). The dotted red lines on the representive slopes and the shaded regions around the trace are SEM. (**b**) Pooled data for the slope of pyruvate production; **** *p* < 0.0001, one way ANOVA with Tukey multiple comparison test. (**c**) Western blot analysis of LDHA and LDHB levels in primary cultured astrocytes expressing LMO, DLDH, and LOx; upper—representative blots; lower—quantification of the ratios of LDHA or LDHB to actin (*n* = 7 for all groups).

Next, we examined the effects of LMO and DLDH on intracellular pyruvate processing in cultured astrocytes using the FRET sensors Pyronic and Laconic. In order to exclude constitutive lactate release, we blocked MCT1 with AR-C155858. Both lactate and pyruvate built up to a plateau, however, the increase in pyruvate levels were still well within the working range of Pyronic. We, therefore, focused on the rate of pyruvate accumulation which was significantly decreased in astrocytes expressing LMO or DLDH, and comparable to the effect of LOx (Figure 7a,b; see [5]). These changes were not attributable to altered expression of lactate dehydrogenase (LDH) as shown by stable protein levels of both subunits LDHA and LDHB in LMO-, LOx- and DLDH-expressing astrocytes (Figure 7c).

## 4. Discussion

Studies of lactate-mediated metabolic and signalling interactions between astrocytes and neurons have lately been held back by the lack of experimental tools to chronically and selectively manipulate the lactate release from astrocytes. To address this issue, we developed viral vector-based molecular tools that take advantage of the superior capacity of bacteria to metabolise lactate.

Both LMO and DLDH are active in many microorganisms where they are involved in glycolysis and fermentation. We selected the LMO sequence from *Mycobacterium smegmatis* and the DLDH from *Lactobacillus delbrueckii* subsp. *bulgaricus* based on their better documented enzymatic activities [15,22]. When adapted to the eukaryotic expression machinery and expressed in mammalian cells, the activity of the foreign enzymes was confirmed. 

A range of factors, such as non-specific side effects of AVV transduction, potential trafficking problems associated with the expression of foreign gene products, interference with glycolytic pathways, as well as by-products of additional enzymatic processes, can all potentially lead to harmful consequences for cells. However, we found that within a normal in vitro working range of AVV MOIs, the expression of LMO and DLDH led to functional enzymatic activity without causing either measurable detriment or signs of apoptotic processes in astrocytes. This was consistent with the expression of the bacterial enzyme LOx which we reported earlier, even though LOx enzymatic activity results in hydrogen peroxide as a by-product, whereas LMO produces acetate, which is expected to be efficiently metabolised further by astrocytes (Figure 1; [5]). In fact, since the data shown here and in [5] were obtained in parallel and are based on the same control experiments, results from the expression of LMO, DLDH, and LOx are directly comparable. Higher levels of LMO, but not DLDH, expression suggested deleterious effects, which may highlight the importance of maintaining basal lactate or NADH levels for astrocytes. The impact of altered astrocytic lactate metabolism on surrounding brain cells, including neurons, should be assessed in future studies.

### 4.1. Lactate Breakdown by LMO

Many studies have pointed out the importance of lactate for CNS metabolism and also, more recently, for brain information processing. Even if it were possible, it would be deleterious for cell and tissue function to eliminate lactate pools too drastically. What our intervention aimed for and achieved was a modulatory limitation that would decrease the amplitude of lactate spikes in response to triggers of astrocytic lactate release [7,14]. These are more likely to play relevant roles in activity-dependent lactate signalling.

As predicted, LMO expression decreased intracellular lactate levels, and this was reflected by a reduction of extracellular lactate concentrations in unstimulated conditions, as well as reduced pool sizes detected with forced pyruvate-induced extrusion. Therefore, in organotypic brainstem slices, we would have expected a reduction in peak lactate release upon trans-acceleration but were not able to detect a significant change. It is, however, important to keep in mind that under these conditions, lactate release was forced from all MCT-expressing cells in the slice culture - including neurons - and LMO was only expressed selectively in astrocytes. Like LMO, LOx also significantly decreased the lactate tone in organotypic slices [5]. In contrast to LMO, however, we observed also a decrease in lactate release upon pyruvate trans-acceleration [5]. Since LMO and LOx exhibit different kinetic properties, with LOx breaking down lactate at approximately a 7000-fold faster rate than LMO, this observation may reflect the faster kinetics of LOx [15]. The astrocyte-selective LMO expression in organotypic slices still significantly affected the maximal drop in lactate release after forced extrusion, and this was the case for LOx expression, as well [5].

We further examined how LMO expression affected the balance of pyruvate and lactate in astrocytes. As expected, when one of the main routes of constitutive lactate export, MCT1, was blocked, lactate accumulated in the cells before pyruvate levels started rising (not shown). This would apply back-pressure on the LDH reaction until an equilibrium between pyruvate and lactate was re-established, consistent with the plateau in the recorded traces. Since the increased lactate levels under these conditions were difficult to quantify accurately, we evaluated the rate of pyruvate accumulation as an indirect measure of lactate build-up when constitutive lactate release was inhibited. The rates of pyruvate accumulation were significantly decreased in LMO-, DLDH- and also LOx-expressing astrocytes and, considering that LDH expression itself remained unchanged, these results are consistent with LMO, LOx, and DLDH activity as an additional lactate sink in the cell.

### 4.2. DLDH

Direct recording of DL production in astrocytes was limited as we had no access to specific biosensors. Laconic is insensitive to DL [19]. We were able to measure DL in conditioned media and indirectly assess the implications of DLDH activity for astrocytic lactate handling via pyruvate detection. Expression of DLDH significantly raised DL release from cells, although the difference with the current version of the construct was modest. Through improvements to the expression system, the yield can potentially be increased in the future.

In unstimulated conditions, in dissociated as well as organotypic slice cultures, lactate release was not significantly affected by DLDH activity in astrocytes. Transient disruption of the lactate/pyruvate/DL equilibrium during trans-acceleration, however, indicated that by diverting pyruvate towards DL synthesis, DLDH had an impact on the maintenance of the steady-state lactate pool.

Endogenous DL production in the brain may occur through the glyoxalase pathway, so the brain must be adequately equipped to process this metabolite. Toxic levels as associated with short bowel syndrome or in the context of diabetic complications cause DL encephalopathy and can interfere severely with brain function [23]. Interestingly, DL has also been suggested as a neuroprotective agent in ischemia [24].

DL shares various molecular targets, such as transporters and receptors, with lactate. For example, MCT1 pumps DL, albeit with a lower affinity and at a slower rate than lactate, or DL activates the lactate receptor HCA1 but with a lower potency than lactate [10,25,26]. To our knowledge, a neurophysiological signalling role of DL has not been described, although DL is known to interfere with memory, possibly through inhibition of cellular respiration or through a receptor-dependent mechanism [27,28,29]. 

With low levels of DL production by astrocytic DLDH expression, we would, therefore, not expect any significant actions of DL via the above targets. We have, however, previously described DL as a potent antagonist which inhibited lactate-induced stimulation of noradrenaline release from locus coeruleus neurons via an unidentified receptor-mediated mechanism [4]. For this target, even moderately increased DL levels, as seen with our DLDH expression system, could potentially have a significant effect.

## 5. Conclusions

In this study, in vitro tests showed that LMO and DLDH are enzymatically active when expressed in mammalian astrocytes and are ready for studies requiring modulation of lactate, production, release and actions in the brain.

## Data Availability

Data reported in this study are available upon request. Clones are available via addgene.org (accessed date 6 August 2021).
